# The use of an O-arm in endonasal endoscopic operations of the skull base

**DOI:** 10.1186/s12893-021-01066-w

**Published:** 2021-01-23

**Authors:** Vlastimil Novák, Lumír Hrabálek, Jan Valošek, Jakub Jablonský, Jiří Hoza, Ivona Korčáková, Martin Hampl, Přemysl Stejskal, Csaba Hučko

**Affiliations:** 1 Department of Neurosurgery, Faculty of Medicine and Dentistry, Palacky University Olomouc and University Hospital Olomouc, I. P. Pavlova 185/6, 779 00 Olomouc, Czech Republic; 2 Department of Biomedical Engineering, University Hospital Olomouc, I. P. Pavlova 185/6, Olomouc, 779 00 Czech Republic; 3 Department of Otorhinolaryngology and Head and Neck Surgery, Palacky University Olomouc and University Hospital Olomouc, I. P. Pavlova 185/6, Olomouc, 779 00 Czech Republic

**Keywords:** Neuronavigation, O-arm, Endonasal approach, Endoscopic skull base surgery

## Abstract

**Background:**

Endoscopic endonasal transsphenoidal approaches are broadly used nowadays for a vast spectrum of pathologies sited in the anterior and middle cranial fossa. The usage of neuronavigation systems (neuronavigation) in these surgeries is crucial for improving orientations deeply inside the skull and increasing patient safety.

**Methods:**

The aim of this study was to assess the use of optical neuronavigation, together with an intraoperative O-arm O2 imaging system, in a group of patients with hypophyseal adenoma that underwent a transnasal transsphenoidal surgery, and correlate the accuracy and its deviation during the navigational process against the use of conventional neuronavigation that uses preoperative MRI and CT scans. The overall group consisted of six patients, between 39 and 78 years old, with a diagnosis of hypophyseal adenoma. Patients were treated with an endoscopic transsphenoidal technique and all of them underwent preoperative MRI and CT scans of the brain. These images were used in the neuronavigation system StealthStation S7^®^ during the surgery, where we defined two bony anatomical landmarks, such as a vomer or the origin of an intrasphenoidal septum, in each operated patient. The tip of the navigational instrument, under endoscopic control, pointed to these landmarks and the distance between the tip and the bony structure was measured on the neuronavigation system. Afterwards, intraoperative 3D x-ray imaging was performed via the mobile system O-arm O2^®^ system with automatic transfer into the navigational system. Under endoscopic guidance, we localized the identical bony anatomical landmarks used in the previous measurement and re-measured the distance between the tip and bony landmark in images acquired by the O-arm. The results of both measurements were statistically compared.

**Results:**

The mean error of accuracy during conventional neuronavigation with usage of preoperative CT and MRI scans was 2.65 mm. During the neuronavigation, with utilization of intraoperative 3D O-arm images, the mean error of accuracy 0 mm. These mean errors of accuracy (both measurement methods were compared by nonparametric Wilcoxon test) had a statistically significant difference (p = 0.043).

**Conclusions:**

Based on this preliminary clinical study, we conclude that the O-arm is capable of providing intraoperative x-ray 3D images in sufficient spatial resolution in a clinically feasible acquisition. The mean error of accuracy during intraoperative navigation, based on 3D O-arm scans at the skull base, is significantly lower compared to the usage of navigation using conventional presurgical CT and MRI images. This suggests the suitability of this method for utilization during endoscopic endonasal skull base approaches.

## Background

Endoscopic endonasal transsphenoidal approaches are broadly used nowadays for a vast spectrum of pathologies sited in the anterior and middle cranial fossa. The usage of neuronavigation systems (neuronavigation) in these surgeries is crucial for improving orientations deeply inside the skull and increasing patient safety. Despite of a lot of evidence in available literature for utilizing neuronavigation during cranial surgeries, like tumor resections or biopsies, so far there is a lack of reports using intraoperative 3D x-ray imaging systems, like the O-arm, in combination with neuronavigation during transsphenoidal approaches. This role is becoming important, especially in situations that need challenging surgical approaches and in situations when anatomical structures are altered by previous surgical or radiological therapy.

Intraoperative visualization of the relationship between a tumor and important neurovascular structures, such as the optic nerve or internal carotid artery (ICA) is essential. One of the most severe complications is an ICA injury, which can result in a potentially fatal outcome. According to the literature, the incidence is between 0 and 3.8% [1, 2]. The usage of neuronavigation that allows the visualization of preoperative scans from magnetic resonance imaging (MRI) and computed tomography (CT) systems decreased the morbidity and mortality rating to under 1% [1]. The aforementioned preoperative scans, however, demonstrate the anatomical conditions before the surgery and do not reflect the current situation in the surgical field. Due to this reason, it is highly desirable to have an intraoperative imaging method that reflects the actual state of the site of the surgery. For this purpose, it is possible to use mobile CT systems or intraoperative MRI scanners in combination with a neuronavigation system. Commonly used neuronavigation systems have a certain degree of deviation in accuracy (mean error of accuracy), which is more frequently detected in deeply localized anatomical structures [3]. These inaccuracies can arise from the registration step during which the neuronavigation’s camera collects points in a 3D coordinate space and fits them onto presurgical CT or MRI images. The parameters of these input presurgical images, like voxel size and slice thickness, or the presence of motion artifacts could also influence inaccuracies in the registration step. On the other hand, utilizing an intraoperative CT scan in combination with neuronavigation allows automatic registration of the acquired scan and rules out possible inaccuracies.

The aim of this study was to evaluate the usage of neuronavigation in cooperation with the intraoperative O-arm O2 system (mobile 3D x-ray system using cone-beam technology), which provides source data in patients with hypophyseal adenoma treated via an endoscopic transsphenoidal approach and to compare its accuracy against conventional neuronavigation using preoperative MRI and CT scans.

## Methods

This prospective pilot study lasted from the 1st of June 2019 until the 31st of October 2019 in the Neurosurgical Clinic of the University Hospital Olomouc. The ethics approval for this study was not required according to national regulations. This study enlisted patients who had operations with a transsphenoidal technique for hypophyseal adenoma. The inclusion criteria for enlisting in the study was the diagnosis of a hypophyseal adenoma indicated for an endoscopic endonasal transsphenoidal approach. The study excluded patients with a former transsphenoidal approach and adenoma recurrence, history of otorhinolaryngologic surgery endonasaly or surgery of paranasal sinuses, inflammatory processes in the nasal cavity, coagulopathy and also patients under the age of 18 years because of the risk of high radiation exposure. The overall group consisted of six patients between 39 and 78 years old (mean age of 64 years old). Out of this, there were four men and two women. Four patients had diagnosis of afunctional hypophyseal macroadenoma. There was one case of hypophyseal macroadenoma with growth hormone hyperproduction (acromegaly) and one case of microadenoma with hyperproduction of adrenocorticotropic hormone (m. Cushing) (Table 1).

**Table 1:** Characteristics of the group

Sex	Age (years)	Preoperative CT	Preoperative CT parameters	Preoperative MRI	Preoperative MRI parameters	Diagnose
Female	78	GE LightSpeed VCT	100 kV, thickness 0.63 mm	Siemens Aera	T1-v, MPRAGE, 1 mm, 192 slices	Macroadenoma-non-functioning
Male	39	GE Discovery CT750	120 kV, thickness 0.63 mm	Siemens Aera	T1-v, MPRAGE, 1 mm, 192 slices	Microadenoma-m. Cushing
Female	73	GE LightSpeed VCT	120 kV, thickness 0.63 mm	GE Signa	T1-v 3D, 1 mm, 176 slices	Macroadenoma-non-functioning
Male	77	GE LightSpeed VCT	120 kV, thickness 0.63 mm	Siemens Aera	T1-v, MPRAGE, 1 mm, 192 slices	Macroadenoma-acromegaly
Male	65	GE LightSpeed VCT	100 kV, thickness 0.63 mm	Siemens Avanto	T1-v, MPRAGE, 1 mm, 192 slices	Macroadenoma-non-functioning
Male	49	GE LightSpeed VCT	120 kV, thickness 0.63 mm	Siemens Avanto	T1-v, MPRAGE, 1 mm, 192 slices	Macroadenoma-non-functioning

**Fig. 1:**
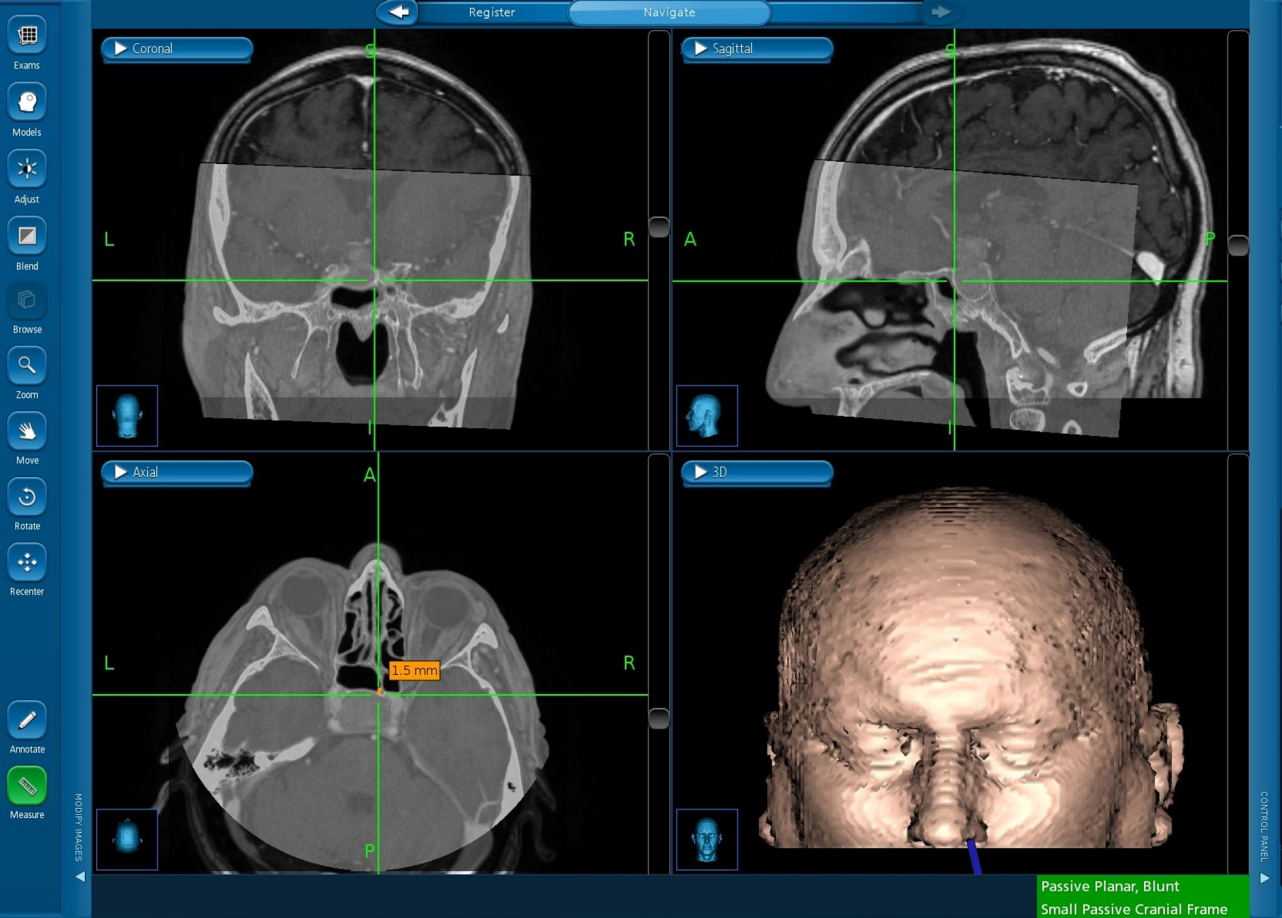
Example of measurement of the distance between pointer’s tip and bony structure (intrasphenoidal septum in this case) on the screen of the neuronavigation system in a presurgical CT scan

Throughout all of the surgeries, optical neurosurgical navigational system StealthStation S7 (Medtronic Navigation Inc., Lettleton, MA, USA) was used for intraoperative navigation with an initial patient head registration performed by the “tracer” method and resulting in an accuracy of < 5 mm. The tracer method is composed of a definition of 3 initial points on the patient’s nose and forehead, followed by a continuous point collection that covers the whole head until the neuronavigation runs out of points. Images from the preoperative 1.5T MR scanner (T_1_-weighted 3D sequence with Gadolinium contrast enhancement, slice thickness 1 mm) and preoperative CT (100 or 120 kV, slice thickness 0.63 mm) were fused together using rigid registration (translation and rotation) through software implemented in the cranial procedure of StealthStation S7 and were used in a mode that allows opacity changes for easy switching between CT and MRI images during surgery. Then, during each surgery, we used two clearly defined, unmistakable bony anatomical structures, such as a vomer or the origin of an intrasphenoidal septum, for defining of anatomical landmarks. The tip of the navigational instrument (pointer) was pointed under endoscopic guidance on these landmarks and the distance between tip of the pointer and bony structure was measured on the screen of the navigation system in CT scans (Fig. 1).

**Fig. 2:**
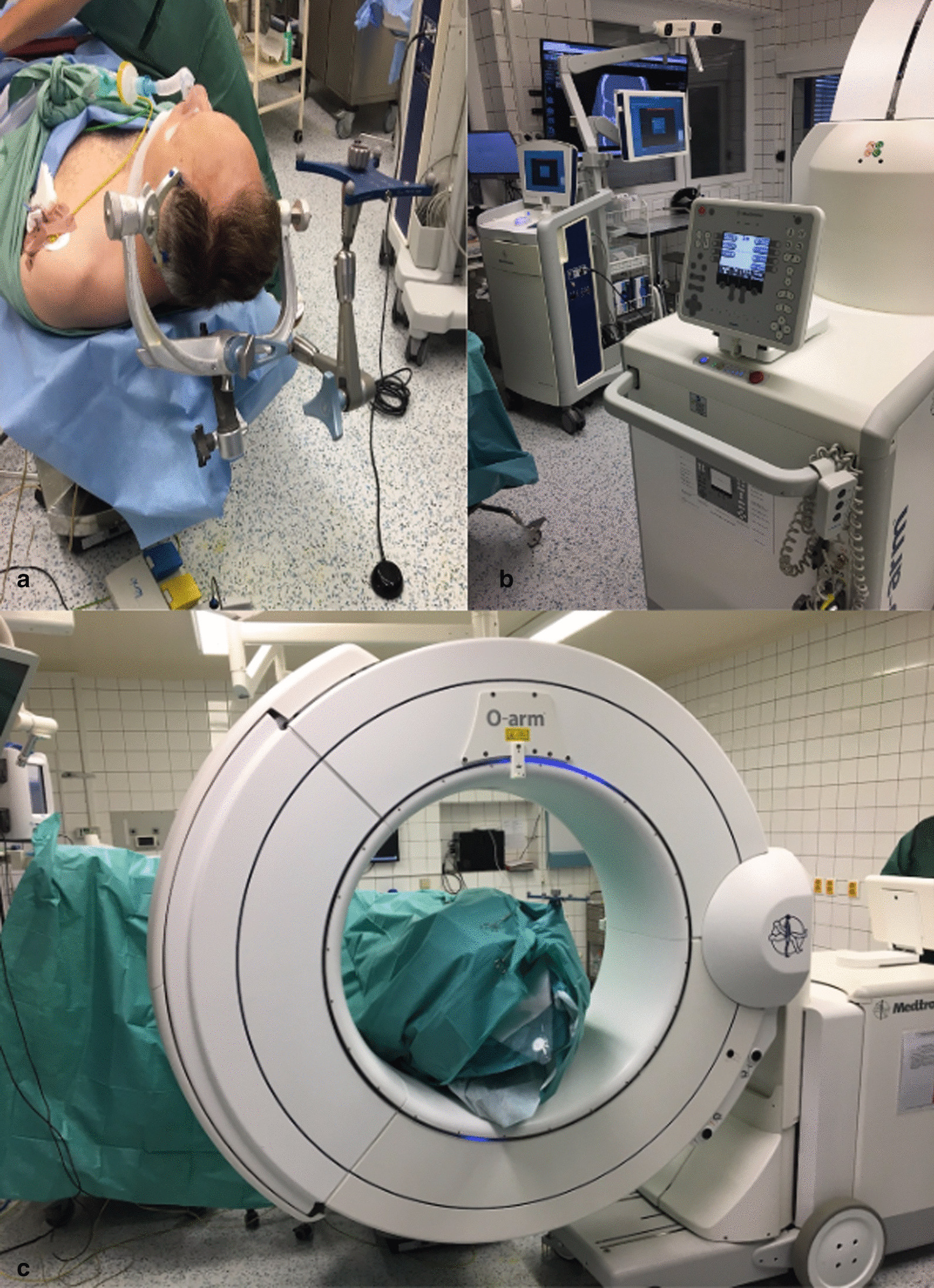
Operating room setting. Patient positioning in the Mayfield skull clamp with a reference frame for neuronavigation (**a**). Setup of a neuronavigation and the O-arm during image acquisition in the surgery room (**b**, **c**)

Afterwards, after opening the sphenoidal sinus and prior to opening the sella turcica and tumor resection, we performed intraoperative scanning using the O-arm O2 system (Medtronic Navigation Inc., Littleton, MA, USA) in the following steps: (1) gantry of the O-arm was set, so the head of the patient was in its isocenter, (2) 2D planar AP and LAT projections were performed for verification of the patients’ head position and adjustment of the gantry, (3) the main 3D imaging was initiated in a stereotactic mode with following parameters: voltage of the X-ray tube = 120 kV, field of view size = 40 cm, voxel size = 0.775 × 0.775 × 0.833 mm, acquisition time < 30 s. During the scanning process, a patient reference frame was attached to the skull clamp and the navigation system’s camera tracked optical markers from both the gantry of the O-arm, as well as the reference frame on the clamp (Fig. 2). An automatic transfer of the obtained 3D scans from the O-arm into the neuronavigation system was performed and the following intraoperative navigation without the need of a manual registration of patient’s head was allowed.

**Fig. 3:**
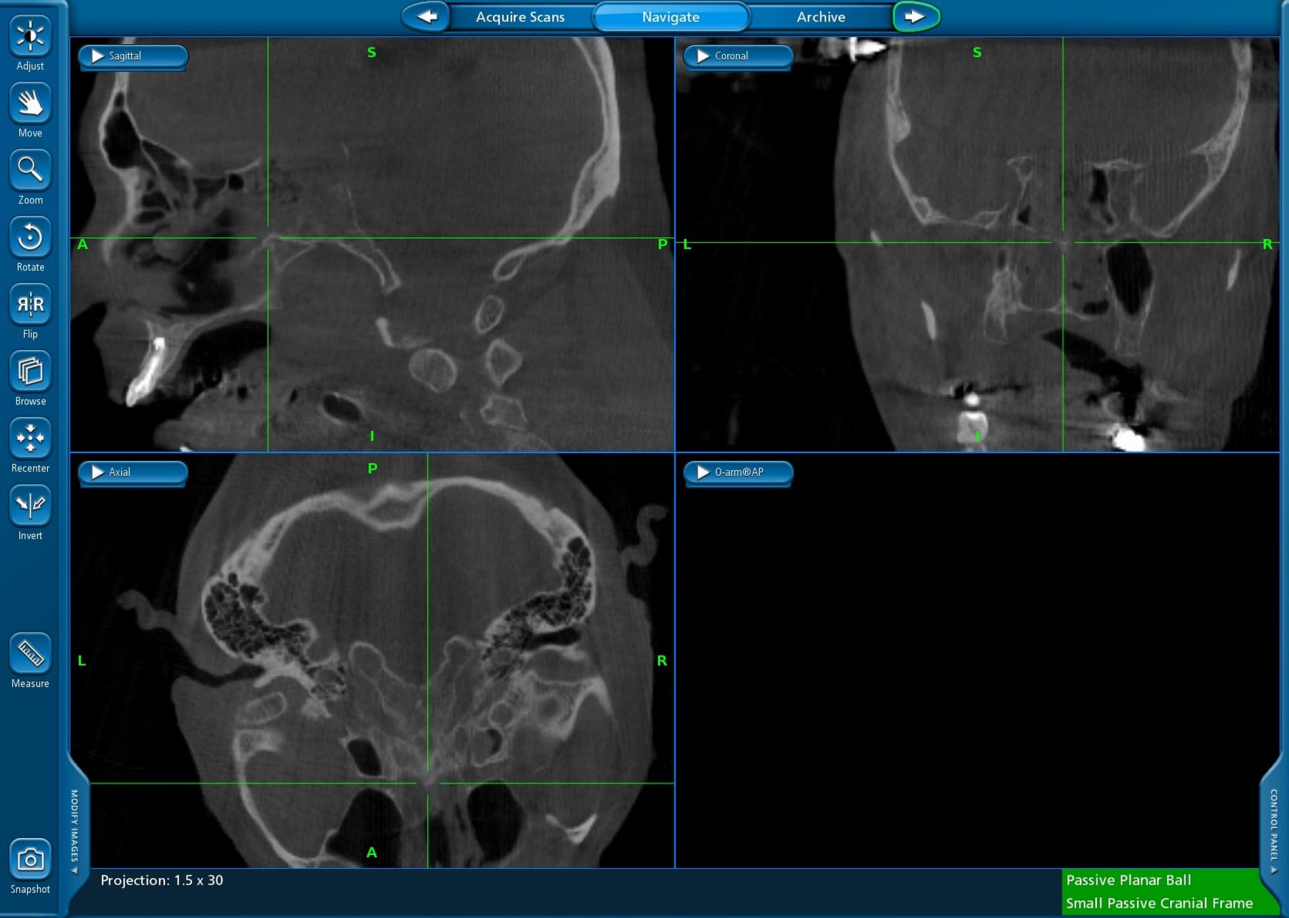
Example of a 3D intraoperative O-arm scan after automatic registration on the screen of a neuronavigation system. The green crosshair indicates the approximate position of the removed anterior wall of the sphenoid cavity

Under endoscopic guidance, we localized the same two anatomical bony landmarks as we did in the first measurement by pointing the tip of the navigational instrument. On the screen of the navigation system, we measured the distance between the tip of the pointer and the bony anatomical landmark in the O-arm images (Fig. 3).

The average absorbed radiation dose during O-arm scanning and the average surgical time was computed.

The data was statistically analyzed by statistical software (IBM SPPS Statistics for Windows, Version 23.0. Armonk, NY: IBM Corp.). In patients, where we measured the deviation in accuracy twice, the resulting measurement was counted by an arithmetic mean. A statistical comparison of both methods was performed by a non-parametric Wilcoxon test (p < 0.05).

## Results

The results of each measurement are shown in Table 2. The mean error in accuracy of the neuronavigation utilizing preoperative CT and MRI scans was 2.65 mm, while the mean error in accuracy of the neuronavigation using intraoperative 3D scans from the O-arm system resulted in 0 mm. Statistical testing of the mean errors of accuracy between each method revealed a statistically significant difference (*p* = 0.043) (Table 3). The average absorbed radiation dose during the O-arm scan was 220.03 mGy cm. The mean surgical time was 104 min.

**Table 2:** Individual measurement parameters for both conventional presurgical CT images and intraoperative O-arm scans

	Reference point	Neuronavigation–accuracy error (mm)	O-arm–accuracy error (mm)	Absorbed dose by O-arm (mGy cm)	Total surgical time (min)
Patient 1	Vomer	6.6	0.0	220.03	125
Patient 2	Intrasphenoidal septum	2.8	0.0	220.03	100
	Intrasphenoidal septum	2.2	0.0		
Patient 3	Intrasphenoidal septum	1.5	0.0	220.03	105
Patient 4	Intrasphenoidal septum	3.6	0.0	220.03	97
	Vomer	3.6	0.0		
Patient 5	Intrasphenoidal septum	0.0	0.0	220.03	66
	Intrasphenoidal septum	0.0	0.0		
Patient 6	Intrasphenoidal septum	1.5	0.0	220.03	130
	Vomer	1.9	0.0

**Table 3:** Statistical comparison of accuracy between neuronavigation and O-arm

*n* = 6	Mean	*SD*	Minimum	Maximum	Median	*p*-value
Neuronavigation-deviation (mm)	2.65	2.27	0	6.6	2.1	0.043
O-arm-deviation (mm)	0	0	0	0	0	

*SD* standard deviation

## Discussion

Early pioneers in transsphenoidal surgery of intrasellar pathologies were very well aware of the crucial need for precise orientation in the surgical field. The historical evolution of imagining method assistance is well known in the literature [4]. In the last 20 years, there has been a significant technological advancement in the field of endonasal skull base surgery. Recent practice is dominated by endoscopic techniques, frameless navigations and intraoperative imaging methods, such as MRI, CT or ultrasound [5].

The combination of an optical neuronavigation system and the O-arm allows intraoperative 3D scans to be acquired, accompanied with automatic registration. This automatic registration rules out possible inaccuracies introduced during manual registration processes used by convectional neuronavigation procedures that are based on preoperative MRI or CT data. Findings that are of this high neuronavigation system accuracy, in combination with 3D O-arm scans proposed in this study, correspond with a previous cadaverous study published by Raza et al. in which a comparison of the accuracy of a navigation system with data acquired from the O-arm in endoscopic endonasal dissections and open approaches targeting the location of middle skull base were performed. The deviation in accuracy was around 0.11 mm till 0.44 mm [6]. An obvious advantage of perioperative 3D O-arm x-ray scans is the clear visualization of bony structures, which is useful in situations where bone anatomy is excessively changed by bone drilling or in places where there is significant osteolysis by the tumor and physiological bony anatomical landmarks are compromised (e.g. invasive macroadenoma or clival chordomas and chondrosarcomas.)

Linsler et al. presented the use of intraoperative high-resolution CT (HRCT) (Siemens SOMATOM Emotion), together, with an optical navigation system in a pediatric patient with hypophyseal macroadenoma. The achieved accuracy was less than 1 mm [7]. This type of intraoperative CT is, however, built-in inside the surgery room and has additional requirements according to the size of surgery room and in utilizing a specialized mobile surgery table that is compatible with this CT. In contrast, the mobile CT systems can be used in existing surgery rooms and can be even easily transported between several surgery rooms. Batra et al. demonstrated usage of mobile intraoperative CT system (xCAT, Xoran Technologies, Ann Arbor, MI, USA) without automatically-registered neuronavigation at the conclusion of endoscopic sinonasal and skull-based surgeries and in 18% of cases they performed additional intervention [8]. However, the automatically-registered navigation using xCAT mobile CT system with submillimeter accuracy (0.34 mm average) has been successfully demonstrated recently on phantoms and could be point of future studies [9, 10]. Till this day, the largest study using a preoperative CT in microsurgical transsphenoidal procedures was published by Eboli et al. The sample consisted of 208 patients. CT images were acquired in the preoperative room and were fused with preoperative MRI scans to allow electromagnetic navigation. However, authors acquired CT images prior to surgery in the preoperative room and not directly during the surgery. Moreover, authors had to perform manual registrations of acquired images using an electromagnetic probe. The resulting accuracy of the navigation was in the range of 1–2 mm. It is also worth mentioning that the radiation dose averaged at around 2000 mGy cm (approximately ten times higher than the one of O-arm) [11].

The positioning of the O-arm in the operating room and image acquisition does not significantly increase the time of surgical procedure. The mean surgical time in our group was 104 min.

The advantage of the O-arm is a significantly faster acquisition of intraoperative scans than in an intraoperative MRI. It even allows easy re-acquiring of images in case of unexpected situations in the operating room (e.g. dislocation of reference frame). Our experiences presented in this study are also being supported by the work of Lauretti et al. In his work, he shares his first experiences with the usage of the O-arm in endoscopic skull base surgeries. He also describes the layout of the equipment in the operating room, acquisition of intraoperative 3D CT scans and its fusion with pre-operative CT and MRI images via neuronavigation [3].

A disadvantage of an O-arm scan is that a patient is exposed to radiation. In our presented study, the absorbed radiation dose of each scan was an identical 220.03 mGy cm. When we compare this number with routinely used head CT scans, where the average absorbed dose peaks are between 500 and 800 mGy/cm, we can conclude that the O-arm dose is significantly lower. Radiation exposure for the staff, if all safety rules are upheld, is near to zero [3, 12]. The main limitation of an O-arm scan is insufficient soft tissue imaging, or more precisely, the inability to appraise the presence of a residual tumor. Improvement of soft tissue imaging and eventually identification of a residual tumor could be achieved by administering a contrast agent. Mori et al. published work using the hybrid operating room with a robotic C-arm used primarily in endovascular procedures. The study was conducted in 12 endoscopically, endonasally operated patients with hypophyseal macroadenoma. After administering the contrast agent, a CT scan was performed, and the presence of a residual tumor was assessed. In nine cases (75%), a residual tumor was identified, and further extirpation carried on [13].

Unfortunately, even after this enhancement, the interpretation would be problematic, especially if the residuum would be located in the vicinity of the cavernous sinus [13]. In this case, the help of an intraoperative MRI is irreplaceable. Unexpected residual tumor detection is, according to the literature, being reported in 15–83% of cases and in 9–83% of cases, the tumor resection continues. Risks of false positive results that can lead to further non-indicated dissection in the operative field still persist. This situation occurs in 2% of cases [5]. So far, intraoperative scanning with ultrasound hasn’t found a wider use in hypophyseal adenoma surgeries [14]. We can see the limits of this study due to the small sample size of our group, which is caused by a low number of patients with pituitary adenoma indicated to transnasal endoscopic surgery. Further prospective clinicals trials are necessary to ultimately delineate the role of this technology in the surgical approaches to the skull base.

## Conclusion

Our preliminary clinical study with the pilot group of patients revealed that the O-arm imaging system, in cooperation with neuronavigation, can provide sufficient spatial resolution, clinically acceptable acquisition time and high accuracy of intraoperative neuronavigation processes during endoscopic endonasal skull base approaches. This finding is confirmed by the mean error of accuracy during O-arm based neuronavigation equal to 0 mm, which is significantly lower compared to navigation utilizing conventional preoperative MRI and CT scans. A lower radiation dose for patients and the possibility of O-arm usage perioperatively also highlight the advantages of the O-arm in comparison to a CT in this type of surgery.

## Data Availability

The data is not publicly available due to individual patient privacy protections.

## Abbreviations

AP: Anteroposterior
CT: Computed tomography
HRCT: High-resolution computed tomography
ICA: Internal carotid artery
LAT: Lateral
MRI: Magnetic resonance imaging
2D: Two-dimensional
3D: Three-dimensional
